# Supplementary material for: Quantitative microscopy of functional HIV post-entry complexes reveals association of replication with the viral capsid
Source: eLife. 2014 Dec 17;3:e04114. doi: 10.7554/eLife.04114 (PMC4293571; doi:10.7554/eLife.04114)
Supplement: Figure 7—source data 1. — Human MDM were infected with NL4-3-R5(IN.eGFP) for 24 hr, fixed, click-labeled, and stained with CC and CA antibodies. RTC/PIC detected were counted from z-stacks covering the whole-cell volume. The table summarizes data from three independent experiments. DOI: http://dx.doi.org/10.7554/eLife.04114.020 [file elife04114s001.docx]

| No. of MDM imaged | | No. of RTC/PIC  detected | Average no. of  RTC/PIC per cell | CA positive RTC/PIC  [%] |
| --- | --- | --- | --- | --- |
| Donor 1 | 19 | 1 | 0.1 | 100 |
| Donor 2 | 82 | 32 | 0.4 | 59 |
| Donor 3 | 53 | 4 | 0.1 | 25 |
| Donor 4 | 29 | 14 | 0.5 | 79 |
